# The influence of negative mood on solitary drinking preference: An experiment with young adult solitary drinkers

**DOI:** 10.1371/journal.pone.0247202

**Published:** 2021-02-18

**Authors:** Carillon J. Skrzynski, Kasey G. Creswell, Timothy Verstynen, Rachel L. Bachrach, Tammy Chung

**Affiliations:** 1 Department of Psychology, Carnegie Mellon University, Pittsburgh, PA, United States of America; 2 Carnegie Mellon Neuroscience Institute, Carnegie Mellon University, Pittsburgh, PA, United States of America; 3 Center for Health Equity Research and Promotion, Mental Illness Research, Education and Clinical Center, VA Pittsburgh Healthcare System, Pittsburgh, PA, United States of America; 4 Department of Psychiatry, Institute for Health, Healthcare Policy and Aging Research, Rutgers, The State University of New Jersey, New Brunswick, NJ, United States of America; University of Queensland, AUSTRALIA

## Abstract

Solitary drinking is a risk marker for alcohol use disorder; thus, it is important to identify why individuals drink alone and for whom this association is particularly relevant. Evidence suggests the desire to ameliorate negative affect (NA) motivates solitary drinking, with some individuals particularly likely to drink alone to cope, but all past studies are cross-sectional. The present study therefore aimed to determine whether 1) experimentally induced NA increased preferences to drink alcohol alone, and 2) whether the relationship between NA and choosing to drink alcohol alone was moderated by neuroticism, drinking to cope motives, and social anxiety. Current drinkers (ages 21-29) with a solitary drinking history (N=126) were randomly assigned to either NA, positive affect [PA], or no affect change (control) conditions via differing cognitive task feedback. After the mood manipulation, participants chose between drinking alcoholic or nonalcoholic beverages in one of two contexts: alone or socially. Evidence regarding effectiveness of the mood manipulation was mixed, and few chose non-alcoholic beverages in either context. Condition did not influence outcome choice. Across conditions, increases in NA and the importance placed on receiving one’s context choice were associated with solitary (versus social) alcohol preference. Neuroticism and its interaction with NA change also influenced choice; individuals high in neuroticism chose more solitary (versus social) drinking contexts while the opposite was true for those low in neuroticism, and among the latter, the preference difference was more pronounced with relatively smaller NA increases. Findings are discussed based on the existing solitary drinking literature.

## Introduction

While most adolescents and young adults drink socially (e.g., [1,2]), about 14% of adolescents [3] and 15-24% of young adults [4] engage in solitary alcohol consumption, most commonly defined as drinking when no one else is present [4]. Though it is less common, solitary drinking may be a risky drinking pattern for younger individuals [5,6]. Indeed, cross-sectional and longitudinal studies demonstrate associations between solitary drinking and a variety of negative psychosocial outcomes in younger individuals. For instance, college students who drink heavily while alone are more likely to report careless and risky behaviors (i.e., missing class, unplanned sex) as well as problems with authorities (i.e., getting arrested because of alcohol or drug use) than their social-only, heavy drinking counterparts [7]. Solitary drinking in young adults is also associated with greater social discomfort measures, including greater social anxiety (e.g., [8–10]) and lower perceived social competence [11]. Recent meta-analytic findings corroborate these findings, with small but reliable effects for the relationship between adolescent and young adult solitary drinking and increased social discomfort (*r*=0.17) [4].

Importantly, emerging evidence suggests that solitary drinking may be an early risk marker for alcohol use disorder (AUD) [12]. For example, solitary drinking in younger individuals is associated with greater quantity and frequency of alcohol consumption (e.g., [10], more negative consequences from alcohol-use (e.g., [13]), greater alcohol dependence severity (e.g., [11]), and greater persistence of problem drinking across time [7]. Of note, longitudinal studies examining solitary drinking have found prospective links from this drinking behavior to later alcohol problems (e.g., [6,12,14]). For example, Tucker et al. (2006) found that 8th grade solitary drinking prospectively predicted alcohol problems at age 23 after controlling for 8^th^ grade alcohol use [6]. Similarly, Creswell et al. (2014) found that drinking alone during adolescence (12-18 years old) predicted DSM-IV-defined AUD symptoms at age 25 after controlling for adolescent alcohol use and problems [12]. Both studies suggest that solitary drinking is an early warning sign for the development of alcohol problems later in life above and beyond other risk factors. Indeed, recent meta-analytic results corroborate these findings, with small effect sizes for the relationships between adolescent and young adult solitary drinking and increased alcohol consumption (*r*=0.23) and more alcohol-related problems (*r*=0.25) [4], highlighting the detrimental aspects of this pattern of drinking and the need to further explore solitary drinking and why it occurs.

The most compelling account for solitary drinking is one of self-medication, in which individuals drink alone to mitigate NA (e.g., [5,12,15]). Indeed, solitary drinking is robustly associated with drinking to cope motives and related factors (e.g., [10,16]). For instance, solitary drinkers endorse drinking to cope with NA more so than social-only drinkers (e.g. [11]), an association that holds even when controlling for social, enhancement, and conformity motives (e.g., [11,17]). Solitary drinkers also report greater beliefs in alcohol’s ability to reduce NA (e.g., [11]), as well as less self-efficacy to resist alcohol when experiencing NA [7], a variable that mediated the relationship between negative emotionality and solitary drinking in a study by Creswell et al. (2015) [15]. Additionally, solitary drinking is associated with both trait and state NA; research has demonstrated links between drinking alone and depression (e.g., [11]), neuroticism/negative affect (e.g., [12,18]), negative temperament [19], as well as associations between drinking alone and state NA such as daily anger and stress [e.g., [18,20]. Finally, solitary drinking is associated with less perceived ability to cope with NA (e.g., [11]). These associations are reliable across studies with meta-analytic findings showing small to medium size effects for the relationships between adolescent and young adult solitary drinking and both a negative affect factor and a negative reinforcement factor including drinking to cope motives and related variables (*r*=0.21 and 0.28, respectively) [4].

Further support for this self-medication model of solitary alcohol use comes from findings demonstrating that PA and drinking for enhancement motives are generally not associated with solitary drinking in younger individuals. For instance, most research has found either a null or negative relationship between drinking alone and social or enhancement motives (e.g., [17,21–23]), as well as no relationship between solitary drinking and drinking in the context of PA (e.g., [12]). Congruent with these findings, meta-analytic results showed that the effect size between drinking alone and a positive reinforcement factor among adolescents and young adults was nearly three times smaller than that of the negative reinforcement factor (*r*=0.10 vs *r*=.28) [4]. Thus, findings converge to support drinking to cope motives as a likely mechanism driving solitary drinking while positive reinforcement is a less probable mechanism.

It is important to note, however, that all studies linking drinking to cope motives to solitary drinking have thus far been correlational in nature, and experimental evidence is lacking. Causal interpretations are thus premature as potential third variables may exist to explain this association, or it may be that solitary drinking induces greater drinking to cope motives. Support for a causal relationship between drinking to cope motives and solitary drinking would have implications for developing effective intervention or prevention programs for young people who engage in solitary alcohol use (e.g., working on developing healthier coping mechanisms and/or targeting individuals who have difficulty with emotion regulation). The present study therefore aimed to determine whether experimentally manipulated NA, in a sample of young adult solitary drinkers, increased the preference to drink alcohol while alone.

If NA increases the preference to drink alcohol while alone in young adults, it will be important to understand for whom this association may be particularly relevant. According to Cooper (1994), negative affectivity or “neuroticism” (i.e. the tendency to experience NA; [24]) should theoretically be related to coping motives given that coping motives are internal and negatively valenced [17]. In fact, neuroticism has been linked to coping motives in young adult samples (e.g., [25]) and, importantly, research has found that the associations between experiences that induced NA (i.e., negative interpersonal exchanges) and drinking alone were stronger for adults higher in trait neuroticism [26]. Additional research found that the relationship between drinking alone and drinking in response to NA was moderated by a social discomfort latent variable including social anxiety, loneliness, and perceived social support [10]. These findings suggest that there may be individual differences (e.g., neuroticism, social anxiety) that increase the likelihood of drinking alone for coping purposes. However, moderation by individual difference factors on negative reinforcement processes has not been thoroughly tested in the literature, let alone in experimental contexts.

This study aimed to address these gaps in the literature by experimentally inducing NA and determining whether NA (versus PA or a no affect change control condition) influenced the type of drinks ordered (i.e., alcoholic or nonalcoholic) and preference for drinking context (i.e., alone or with an ostensible other participant) in a sample of young adults (N=126) with a history of drinking alone. Individuals randomly assigned to the NA condition were hypothesized to be more likely to choose alcoholic (versus nonalcoholic) beverages and to choose to drink these beverages alone (versus with an ostensible other participant) compared to individuals randomly assigned to a PA or a no affect change (i.e., control) condition. Individual differences in neuroticism, drinking to cope motives, and social anxiety were hypothesized to act as moderators, such that an increase in these variables would magnify the relationship between NA and choosing to drink alcohol alone. The design for this study, as well as power analyses, proposed hypotheses, and the data analytic plan were pre-registered on Open Science Framework before data collection began (https://osf.io/e7yxn/register/5771ca429ad5a1020de2872e).

## Materials & methods

### Participants

Male and female current drinkers (N=126, 57.9% females) were recruited from the Pittsburgh area via local flyers and advertisements in a local research registry (i.e., Pitt + Me) with the cover of investigating how alcohol influences memory recall for cognitive and social experiences (despite no actual alcohol consumption in the study—see materials and procedure below). Advertised duration of the study was between 1.5 to 2 hours, and compensation was $20. Participants were eligible if they were between the ages of 21 and 29, drank alcohol at least once per week, had a history of solitary drinking (i.e., endorsed that at least 25% of their time spent drinking in the past 6 months was done while no one else was present), had no prior exposure to alcohol administration studies, and were not pregnant or nursing if female (to enhance the belief that alcohol would be consumed). All procedures for the study were approved by the Carnegie Mellon University IRB, and all participants provided informed consent.

### Procedure and measures

A series of pilot studies (with a total of 136 participants) were conducted to refine procedures for the familiarity task and mood manipulation (see below) prior to enrolling participants for the experiment. Participants for the current study arrived in the laboratory between the hours of 12:00 and 4:00 p.m. At the beginning of the session, they completed baseline questionnaires assessing demographic variables, alcohol use variables (e.g., frequency and quantity of past year alcohol consumption), drinking motives, social variables (e.g., social anxiety), and personality variables (e.g., neuroticism). Afterwards, participants engaged in the familiarity task.

#### Familiarity task

A familiarity task was used to mitigate the concern that non-familiarity with the ostensible drinking partner would have a potential confounding effect on drinking context preference (i.e., participants might not want to drink with a complete stranger). Participants were asked to read a personal description of a fictitious character who they were told was another participant present in the lab. Participants also wrote their own personal description based on a list of prompts under the guise that the ostensible other participant was going to read theirs. To mitigate any potential suspicions about the task, participants were told that they would be asked questions about each other’s descriptions after drinking alcohol, for the purpose of examining how alcohol influences social memories.

The familiarity task was a modified version of the Relationship Closeness Induction Task [27], which includes personal questions (e.g., favorite season) that participants share with one another. Because the goal was to increase familiarity without inducing significant social bonding, which can occur in face-to-face interactions (as observed in two of the pilot studies we conducted, mentioned above), we used this modified version of the task that relied on written descriptions. The personal description the participants read of the ostensible other participant was pre-scripted with gender-neutral information based on population data. For example, one statement was, "My favorite color is blue" because blue is the most popular color among both men and women [28].

#### Mood manipulation

Participants were randomly assigned to one of three conditions (i.e., NA, PA, or a no affect change [i.e., control condition]) via a performance manipulation involving the Remote Associates Test (RAT; [29]), as has been used in other studies (e.g., [30,31]). This manipulation was chosen because it was likely to induce self-relevant NA, which seems to be particularly important in driving solitary alcohol consumption [26,32,33], rather than using a manipulation that induces general NA (e.g., sadness related to an external event such as viewing a sad film clip). Specifically, all participants completed 3 difficult and 7 easy items from the RAT. These items consisted of three words that had one word in common; participants needed to supply this fourth word to complete the items (an example of a difficult item used was “widow,” “bite,” and “monkey”, which are all related to the word “spider”, the correct answer). Participants were told that the RAT is a globally used intelligence test which is a valid and reliable predictor of future success, and that they were completing it to create cognitive memories in order to determine how alcohol affects recall of such memories. After participants were finished, the experimenter scored the answers for all participants in a separate room. When the experimenter returned, they told participants randomly assigned to the NA condition that their scores were below average, and they were in the bottom 20th percentile of participants in the study. Participants randomly assigned to the PA condition were told their scores were above average, and they were in the top 20th percentile of participants in the study. Participants randomly assigned to the control condition were given no feedback and were not aware that their tests had been scored. [Note: This decision was based on the pilot testing mentioned above, in which control participants were originally told their score was in the same range as other individuals in the study; this led to reported increases in both PA and NA. After piloting a no feedback control condition, participants displayed no increase in either affect.].

#### Manipulation check

Eight items from the Positive and Negative Affect Schedule (PANAS; [34]) were used to test whether the affect manipulation was effective in inducing the desired affect (or lack of change in affect) in the respective conditions. The PANAS was completed before and after the mood induction. Participants rated their present moment positive and negative affective states using four negatively-valenced (i.e., upset, troubled, frustrated, worried/anxious) and four positively-valenced (i.e., excited, interested, pleased, joyful) items with options ranging from 1(very slightly or not at all) to 5(extremely). Responses on the negatively-valenced and positively-valenced items were summed to create NA and PA scores used in analyses. The PANAS has been used to successfully detect changes in affect in a prior study that implemented the RAT as a mood manipulation [35]. Additional measures that assessed participants’ enjoyment and liking of the ostensible other participant were administered after the PANAS to ensure that such variables did not confound drinking context preference. Participants were asked items such as, “How much did you enjoy reading the other participant’s personal description?” and “How much do you like the other participant?” with response options ranging from 1(not at all) to 7(a great deal).

#### Primary outcome

After completing the PANAS following the mood manipulation, participants were asked whether they wanted to drink alcoholic or nonalcoholic beverages, and to indicate which room they preferred to consume their beverages in while the next task was being set up in their currently occupied room. Specifically, the experimenter stated, “In a few minutes we’ll begin the drinking portion of the study. We need several conditions in this study to test how alcohol influences social and cognitive memories, including a comparison condition in which non-alcoholic beverages are consumed. We’re letting participants choose which condition to be in. You will be consuming a beverage shortly, but this can either be an alcoholic or non-alcoholic drink. We have a wide range of beer, liquor, and mixers, as well as a wide range of sodas, teas, and juices for you to choose from, so we’re confident you’ll find something you like. I’ll get your exact drink order right before we begin drinking, but because I need to set up the final task in this room while you’re drinking, I’ll have you consume your beverage in another room. You can either drink in this room [point] (the other participant’s in there) or in this room [point], which is empty.” After this, we asked participants about their drink and room choices, the order of which was counterbalanced across participants: Order 1: “So, which room do you prefer to drink in and would you prefer an alcoholic or nonalcoholic beverage?” or order 2: “So, would you prefer an alcoholic or nonalcoholic beverage and which room do you prefer to drink in?” The answers the participant gave were recorded on an experimental note sheet and used as the dependent variable in analyses. Following this question, participants completed a second set of questionnaires (see importance variables below), were debriefed, given compensation, and dismissed. Participants did not actually receive a beverage during the session.

### Individual difference variables

Three questionnaires administered at baseline were used to examine the moderating influence of neuroticism, drinking to cope motives, and social anxiety. [Note: These variables were not included as eligibility criteria, rather we assessed naturally occurring variation on these variables in a sample of solitary drinkers]. Neuroticism was measured with the neuroticism subscale of the Neuroticism-Extraversion-Openness Five Factor Inventory-Revised version 3 (NEO-FFI; [24]), which has 12 items with responses ranging from 0(disagree strongly) to 4(agree strongly). An example item is “When I’m under a great deal of stress, sometimes I feel like I’m going to pieces”. Drinking to cope motives were assessed via the drinking to cope subscale from the Drinking Motives Questionnaire-Revised (DMQ-R; [17]). Individuals are instructed to indicate endorsement for five items related to drinking to cope motives (e.g., “To forget your worries”) with response options ranging from 1(almost never/never) to 5(almost always/always). Social anxiety was measured with the Social Interaction and Anxiety Scale (SIAS; [36]), which assesses fear of social interactions with 19 items (e.g., “I have difficulty talking with other people”) ranging from 0(not at all) to 4(extremely). Responses on the neuroticism and SIAS scales were summed and responses on the DMQ-R drinking to cope subscale were averaged for final scores to be used in analyses. All three scales demonstrated adequate to high reliability (α=0.82, 0.83, 0.94 for neuroticism, DMQ-R and SIAS, respectively).

### Importance variables

Two variables were included to assess the importance participants’ placed on receiving their choice of drink (i.e., alcoholic or non-alcoholic) and context (i.e., alone or with the ostensible other participant). These were measured with two questions: “How much do you care about whether you get the type of drink you picked?” and “How much do you care about whether you do the rest of the experiment alone or with someone else?” with numbered options from 1(not at all) to 5(very much). These variables were included as control variables in all analyses, as well as examined as moderators to see if the relationship between NA and choosing to drink alcohol alone would be magnified by the importance participants placed on receiving this choice. Their inclusion as control variables at the start of model testing was done to account for the fact that some individuals might make their choices more capriciously than others regardless of condition.

### Power analysis

An a priori power analysis was conducted to determine the sample size needed to detect a small effect size. Data was simulated in R [37] by creating condition columns (NA, PA, and control—dummy coded), an error term (Gaussian distribution with a mean of 0 and standard deviation of 1), and a choice column designating the four possible outcomes (1=alcoholic beverage + alone, 2=non-alcoholic beverage + alone, 3=alcoholic beverage + with other, and 4=non-alcoholic beverage + with other). The choice column was based on probabilities for cells 1 and 3 equating to small odds ratios (~1.4); for the NA condition, this was equal to a 59% chance of selecting choice 1 (0.59/(1-0.59)=1.44) and a 24% chance of selecting choice 2 (0.24/((1-0.59)-0.24)=1.41). This was reversed for the PA condition (59% for choice 3 and 24% for choice 1). The remaining probabilities (1-0.83=0.17) were split between choices 2 and 4. Because we didn’t have specific hypotheses for the control condition, all 4 choice outcomes had a 25% chance of being selected. Using these probabilities, a Monte Carlo simulation was conducted in which 10,000 multinomial logistic regressions were run to see how many significant p-values emerged per comparison (i.e., 1 compared to 2, 1 compared to 3, and 1 compared to 4) depending on different sample sizes. Using an alpha of 0.05, power of 0.80, and a small effect size (odds ratio=1.4), 150 participants were required (50 participants per condition). Due to the COVID-19 pandemic and closure of the Carnegie Mellon University campus, only 128 participants were run. Two participants were excluded before data analyses were conducted because they did not meet eligibility requirements during the experimental session, despite meeting requirements during the phone screen (i.e., they reported past solitary drinking during the phone screen, but denied past solitary drinking during the experimental session). Thus, the final sample included 126 individuals.

## Data analysis

### Preliminary analyses

Preliminary analyses were conducted to ensure that randomization of participants to the three conditions was successful. Analyses (e.g., ANOVA, chi-square) were run to identify any significant differences across the three conditions on the following variables: demographics, alcohol consumption variables, neuroticism, social anxiety, drinking motives, familiarity/liking of the ostensible other participant, scores on the RAT, and importance of getting their drink and room choices. Variables that significantly differed between conditions were included as covariates in the primary analyses in addition to the importance variables.

### Manipulation check

Following the preliminary analyses, two sets of analyses were run to ensure the mood manipulation was effective in increasing NA (and decreasing PA) in the NA condition, increasing PA (and decreasing NA) in the PA condition, and generating no change in affect in the control condition. The first set was carried out using repeated measures analysis of variance (ANOVA) tests with time of PANAS scores (i.e., pre- and post-RAT) as a within-subjects factor. Separate models were run for each condition and for NA and PA. The second set used analysis of (co)variance (ANCOVA) to examine whether post-RAT NA differed across conditions controlling for pre-RAT NA. These tests were also run for PA. For each ANCOVA, post hoc multiple comparisons tests were generated to assess which conditions differed significantly from each other on NA and PA.

### Primary analyses

#### First hypothesis

Following the manipulation check, primary analyses were conducted to assess the two main hypotheses—that individuals in the NA condition would be more likely to choose to drink alcohol alone, and that individuals in the NA condition who were high on trait neuroticism, drinking to cope motives, and/or social anxiety would be especially likely to choose alcohol to drink alone. A series of multinomial logistic regression (MLR) analyses were run in R [37] using the function “multinom” via the nnet package [38]. For all analyses, the dependent variable was the overall choice made by each participant from the four possible options (i.e., 1=alcoholic beverage + alone, 2=non-alcoholic beverage + alone, 3=alcoholic beverage + with other, 4=non-alcoholic beverage + with other), and the reference category for all models was the alcoholic beverage + alone option, which was compared to the three other options. To aid in interpreting the results, dummy codes were created for the conditions such that 0=not in the condition and 1=in the condition. Thus, for each of the three condition dummy codes, beta coefficients designated the increase (or decrease) in the log odds of selecting one of the three outcomes compared to drinking alcohol alone as individuals went from not being in that condition to being in that condition.

#### Second hypothesis

To test the second hypothesis of moderation, interaction terms were created for social anxiety, drinking to cope motives, and neuroticism. Originally, these interactions terms were to be created using the NA condition dummy code, but manipulation checks showed that (unexpectedly) the control condition also experienced an increase in NA in addition to the NA condition. Thus, an NA change score was created to examine whether increases in NA, regardless of condition assignment, increased the likelihood of choosing to drink alcohol while alone (see [39] for a discussion of the strengths of using such change scores in repeated measure designs). Because the condition variables were not retained in subsequent model testing (because condition did not influence outcome choice) while NA change was (see below regarding variable selection), NA change was used in all interaction terms to assess for moderation. Interaction terms were made by multiplying NA change (a continuous variable) by the continuous variables of social anxiety, neuroticism and drinking to cope motives. To be comprehensive, a PA change score was also created to test as a potential predictor. These analyses, using NA and PA change scores, should be considered exploratory since they were not included in the analyses proposed in the preregistration report.

#### Feature selection – base model regarding first hypothesis

A feature selection analysis was performed, using stepwise model selection, to establish which variables (i.e., condition, NA and PA change score, and moderating variables) were the strongest predictors of outcome choices. Methods such as this, that employ more nuanced approaches to statistical analyses over traditional null hypothesis testing, have been recommended by the American Statistics Association (see [40]). Models were run in two phases in stepwise fashion. The first step was used to establish whether condition, NA change, and/or PA change should be retained (i.e., whether they influenced outcome choice) in regards to the first study hypothesis. This was done through first running a null model (i.e., a model that only included an intercept term), as well as four alternative base models that included condition, NA change, PA change, and their combinations in predicting outcome choice (i.e., 1: a model with just condition, 2: a model including condition, NA change, and PA change, 3: a model including both NA and PA change, and 4: a model with just NA change). These models were compared with Akaike’s Information Criterion (AIC; [41,42]). The AIC is a relative index of model fit such that a model with a smaller AIC (relative to another model AIC) is a better fitting model [43]. Five thousand bootstrapped AICs were obtained per model through the “boot” function in R [44], and 95% confidence intervals (CIs) were generated for the differences between these bootstrapped AICs for each pair of models (e.g., a 95% CI was produced for the difference between the bootstrapped null model’s AICs minus the bootstrapped AICs of an alternative model, another 95% CI was produced for the difference between that alternative model’s bootstrapped AICs minus another bootstrapped model’s AICs). Because the null model contained only the intercept (i.e., the mean of the outcome) it is assumed to have good fit to the data from the outset. Therefore, when alternative models produced smaller AICs than the null model, they were retained as better fitting models. After comparing these alternative models to each other and finding the one that produced the smallest AICs, a final base model was selected.

#### Feature selection – moderation model regarding second hypothesis

Following selection of the best fitting base model, a second step was implemented such that the three individual difference moderator variables (i.e., neuroticism, drinking to cope, and social anxiety) and interaction terms (e.g., neuroticism x NA change) were each added to the base model to produce 3 more models for comparison, and the same procedure was followed to select a final best fitting model from these. Finally, the best fitting model was compared to one that also tested for moderation by the importance participants placed on receiving their context choice as well as one that tested for moderation by the importance participants placed on receiving their drink choice (i.e., context importance x NA change; drink importance x NA change).

#### Model exploration

After finding the best fitting moderation model, beta coefficients from this model were submitted to bootstrapping, as well as the resulting 95% CIs, to investigate the influence of the included variables on the outcome. This was done to determine the magnitude and confidence of individual effects, rather than assessing significance via p-values, given that the stepwise selection process already identified the necessary model features that best explained variance in outcome (otherwise this combination of variables would not result in the best fitting model; e.g., [45]). The bootstrapped coefficients were also plotted as histograms to view their distributions and assess whether coefficients fell primarily to either side of zero. Likewise, z-scores of the distance between 0 and the mean of the bootstrapped coefficients, as well as percentages of coefficients that were above or below zero (depending on the sign of the coefficient), were investigated. This was done to examine the relationships of the variables to the outcome in a more comprehensive way.

## Results

### Descriptive statistics and bivariate correlations

Descriptive statistics are shown in Table 1 and bivariate correlations between variables in Table 2. As shown in Table 1, randomization was successful in that conditions did not vary significantly on any variable. On average, participants (59.6% female) were 24.44 years of age (*SD* = 2.70), consumed 2.98 drinks per occasion on an average of 2 to 4 days per week, and spent 47.61% of their time drinking alcohol in the last 6 months alone. The majority of individuals were Caucasian (61.9%), while 15.9% were African American, 15.9% were Asian, 0.8% were Native Hawaiian or other Pacific Islander, 1.6% were American Indian/Alaskan Native, and 4% were more than one race. As shown in Table 2, solitary drinking was positively associated with drinking frequency and drinking to cope motives, though it was not related to alcohol quantity. It was also negatively associated with RAT scores. Not surprisingly, neuroticism, drinking to cope motives, and social anxiety were positively associated with each other, and both neuroticism and social anxiety were positively associated with NA change. NA change was negatively associated with PA change. PA change was negatively associated with drinking to cope motives but nothing else. The importance participants placed on receiving their drink choice (i.e., drink importance) was positively associated with the importance participants placed on receiving their context choice (i.e., context importance), but the association was weak indicating that these variables should be examined separately. While drink importance was not related to any other variable, context importance was positively related to neuroticism, drinking to cope motives, and social anxiety. Drink choice (i.e., alcohol or non-alcohol) was positively associated with NA change and negatively associated with typical alcohol frequency, such that as NA increased and alcohol frequency decreased, participants were more likely to choose a non-alcoholic beverage. Finally, context choice (i.e., alone or social) was negatively associated with drink choice, social anxiety, context importance, and positively associated with typical quantity of alcohol consumption. Participants were more likely to choose to be alone if they chose a non-alcoholic beverage, as well as if social anxiety and the importance they placed on receiving their context choice increased, and quantity of typical alcohol consumption decreased.

**Table 1:** Descriptive statistics for variables across condition.

	NA Condition	PA Condition	Control Condition		
	N=44	N=39	N=43		
Variable	*Mean* (*SD*)/%			*F/Χ*^*2*^	*p-* value
Age	24.68 (2.76)	24.41 (2.88)	24.21 (2.51)	0.33	0.72
Sex				0.90	0.64
Female	52.3	61.5	60.5		
Male	47.7	38.5	39.5		
Marital Status				6.14	0.19
Married	6.8	0.0	11.6		
Engaged	6.8	2.6	2.3		
Single	86.4	97.4	86.0		
Education^1^				2.30	0.68
High school	11.4	5.1	4.7		
Undergraduate	56.8.2	61.5	67.4		
Graduate	31.8	33.3	13.3		
Occupation				2.19	0.70
No job	25.0	28.2	27.9		
Part time job	38.6	43.6	30.2		
Full time job	36.4	28.2	41.9		
Race				7.16	0.71
White	56.8	64.1	65.1		
Asian	20.5	10.3	16.3		
African American	18.2	17.9	11.6		
Native Hawaiian/Pacific Islander	0.0	2.6	0.0		
American Indian/Alaska Native	2.3	2.6	0.0		
Multiracial	2.3	2.6	7.0		
Typical Frequency	3.98 (1.66)	3.87 (1.54)	3.81 (1.42)	0.13	0.88
Typical Quantity	3.18 (2.13)	3.08 (2.28)	2.67 (1.42)	0.80	0.45
Solitary Drinking	42.55 (19.71)	44.56 (21.51)	43.67 (22.63)	0.09	0.91
Neuroticism	40.52 (9.39)	37.05 (10.13)	39.77 (9.22)	1.48	0.23
SIAS Total	29.05 (16.87)	24.15 (17.46)	28.60 (14.42)	1.12	0.33
Drinking to Cope Motives	2.29 (0.91)	2.12 (0.94)	2.08 (0.79)	0.71	0.49
RAT Score	5.05 (1.98)	5.23 (2.24)	6.00 (1.88)	2.67	0.07
Liked writing description	4.41 (1.45)	4.46 (1.27)	4.40 (1.40)	0.03	0.97
Liked reading description	5.18 (1.17)	5.21 (1.08)	5.05 (1.34)	0.21	0.81
Familiarity with other	4.91 (1.31)	4.36 (1.29)	4.42 (1.16)	2.43	0.09
Perceived Interaction Enjoyment	5.23 (1.21)	4.86 (1.31)	5.14 (1.39)	0.90	0.41
Liking of other	4.91 (1.21)	4.87 (0.95)	5.00 (0.95)	0.17	0.85
Drink Importance	2.64 (1.35)	2.21 (1.32)	2.09 (1.02)	2.33	0.10
Context Importance	2.57 (1.42)	2.23 (1.33)	2.23 (1.33)	0.91	0.41

^1^ Education was assessed as the highest grade level a participant had completed and was recoded such that individuals were categorized into those whose highest grade achieved was during high school, during undergraduate training, or during graduate training.

Note. Typical frequency=past year drinking frequency; typical quantity=past year typical drinking quantity; solitary drinking=percent of lifetime solitary drinking; SIAS=social interaction anxiety scale; RAT=remote associates task score; drink importance=importance placed on receiving one’s drink choice; context importance=importance placed on receiving one’s context choice.

**Table 2:** Bivariate correlations between variables of interest.

	1	2	3	4	5	6	7	8	9	10	11	12
1. Frequency	-											
2. Quantity	0.34**											
3. Solitary	0.30**	0.08	-									
4. Neuroticism	0.16	0.24**	0.07	-								
5. SIAS	-0.01	0.11	-0.00	0.56**	-							
6. Coping	0.20*	0.34**	0.26**	0.62**	0.37**	-						
7. RAT	0.12	-0.09	-0.34**	0.12	0.12	-0.08	-					
8. Drink Import	0.05	0.03	0.08	0.10	0.10	0.09	-0.09	-				
9. Context Import	-0.04	0.06	0.03	0.25**	0.38**	0.22*	0.10	0.23**	-			
10. NA Change	-0.05	0.07	-0.01	0.21*	0.18**	0.13	-0.15	0.05	0.09	-		
11. PA Change	-0.14	-0.10	0.01	-0.10	0.00	-0.24**	0.16	-0.03	-0.04	-0.35**	-	
12. Drink Choice	-0.16	-0.20*	-0.06	-0.07	-0.00	-0.10	-0.11	-0.03	0.08	0.21*	0.01	-
13. Cont Choice	0.18*	-0.05	0.04	-0.11	-0.29**	-0.04	0.04	0.01	-0.44**	0.00	0.05	-0.18*

p < 0.001** p<0.01*.

Note. Frequency=past year drinking frequency; quantity=past year typical drinking quantity; solitary=percent of lifetime solitary drinking; SIAS=social interaction anxiety scale; coping=drinking to cope subscale of DMQ-R; RAT=remote associates task score; drink import=importance placed on receiving one’s drink choice; context import=importance placed on receiving one’s context choice; drink choice=the choice between alcohol or non-alcohol (0=alcohol, 1=non-alcohol); cont choice=the choice between drinking preferred beverage alone or socially (0=alone, 1=social).

### Manipulation check

Table 3 depicts the PA and NA pre-RAT and post-RAT mood ratings (mean, SD) across conditions. Consistent with expectations, there was a significant main effect of time on both NA and PA for the NA condition, *F*(1,43)=28.71, *p*<.001 and *F*(1,43)=31.05, *p*<.001, respectively, such that NA significantly increased, and PA significantly decreased, from pre- to post-RAT. Contrary to expectations, there was no change in PA from pre- to post-RAT in the PA condition, *F*(1,38)=0.60, *p*=0.45, nor was there a change in NA, *F*(1,38)=2.12, *p*=0.15. Also unexpectedly, there was a significant main effect of time on NA for the control condition, *F*(1,42)=4.72, *p*=0.04, such that NA significantly increased from pre- to post-RAT, though there was no change in PA, *F*(1,42)=0.63, *p*=0.43.

**Table 3:** Mood ratings (mean, SD) across time points and conditions.

Condition	Pre-RAT PA	Post-RAT PA	Pre-RAT NA	Post-RAT NA	NA Change	η^2^	PA Change	η^2^
NA	11.30 (3.81)	9.84 (4.05)	5.61 (2.14)	7.98 (3.30)	2.36 (2.93)	0.40	-1.45 (1.73)	0.42
PA	11.46 (3.42)	11.72 (3.97)	4.77 (1.18)	5.15 (1.86)	0.38 (1.65)	0.05	0.26 (2.07)	0.02
Control	11.58 (3.40)	11.35 (3.72)	5.30 (1.46)	6.09 (2.52)	0.79 (2.39)	0.10	-0.23 (1.93)	0.02

Further analyses demonstrated potential differences between conditions on initial (i.e., pre-RAT) NA (*F*(2,123)=2.72, *p*=0.07), but not pre-RAT PA (*F*(2,123)=0.07, *p*=0.93). Post-hoc multiple comparisons revealed that the NA condition had greater pre-RAT NA than the PA condition (*p*=0.02) but not the control condition (*p*=0.38), while the PA and control conditions did not differ from each other on pre-RAT NA (*p*=0.15). Because of this significant baseline difference, an ANCOVA comparing post-RAT NA was run, controlling for pre-RAT NA across conditions. As shown in Table 4, results were statistically significant. Consistent with expectations, post hoc multiple comparisons revealed that, controlling for pre-RAT affect, post-RAT NA was significantly greater in the NA condition compared to both the PA and control conditions, and did not significantly differ across the PA and control conditions.

**Table 4:** ANCOVAs of post-RAT NA and PA controlling for pre-RAT NA with multiple comparisons across conditions.

	Post-RAT NA	
	F	p
ANCOVA	21.04	<0.001
Multiple Comparisons		
NA vs PA		<0.001
NA vs Control		<0.01
PA vs Control		0.30
Adj. Means	M(SE)	
NA	7.71 (0.36)	
PA	5.50 (0.39)	
Control	6.05 (0.36)	

Note. Adjusted means=means of post-RAT affect after controlling for pre-RAT affect.

### Primary analyses

**Fig 1:**
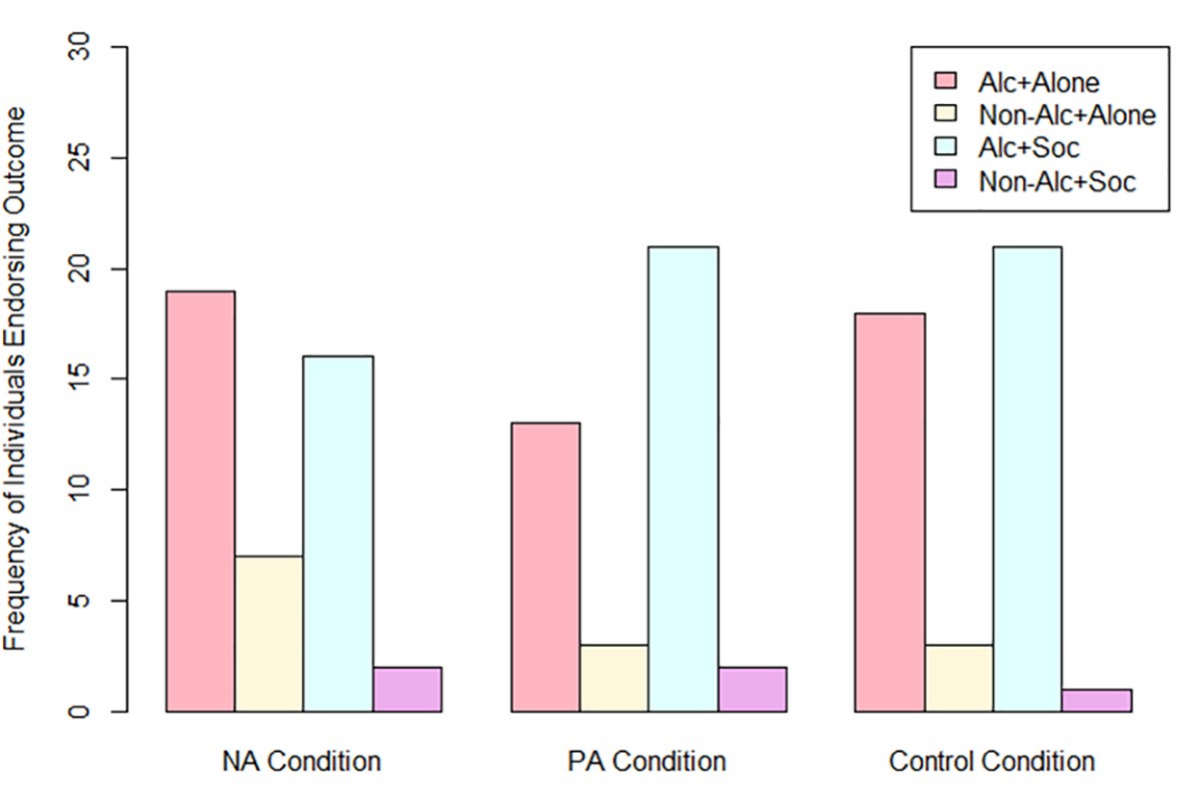
The number of individuals per condition who chose each of the four outcomes. Alc+Alone=choosing to drink alcohol while alone; Non-Alc+Alone=choosing to drink non-alcohol while alone; Alc+Social=choosing to drink alcohol with the ostensible other participant; Non-Alc+Social=choosing to drink non-alcohol with the ostensible other participant.

Fig 1 displays outcome choices across conditions. Descriptively, outcome varied by condition such that more individuals in the NA condition chose to drink alcohol alone (N=19) relative to drinking alcohol socially (N=16), compared to the PA and control conditions; in both of these conditions, more individuals chose to drink alcohol socially (N=21 for both conditions) than drink alcohol alone (N=13 and 18 for the PA and control conditions, respectively). Few individuals in any condition chose to drink non-alcoholic beverages in either context (see Fig 1).

### Model selection

#### Step 1: Base model selection – First hypothesis

As described above, feature selection was implemented to initially see which variables should be retained in the base model (i.e., controlling for the importance participants placed on receiving their choices, whether condition, NA change, and PA change should be included together and/or if certain combinations of these variables resulted in a better fitting model) and then following this, which moderation variables should be included. Table 5 shows which variables were included in each model that was tested along with corresponding AIC values, while Table 6 shows 95% CIs of the differences between bootstrapped AICs for every pair of models. Based on the 95% CIs, all base alternative models (models 1-4, see Table 6) produced a better fit to the data than the null model; that is, all four generated smaller AICs than the null model. Further, models 2-4 produced smaller AICs compared to model 1, but there was inconsistent evidence that models 2-4 were better fitting models than each other when comparing the 95% CIs. Given this inconsistency, the simplest model that only contained NA change (i.e., model 4) was chosen as the base model to move forward. Thus, condition and PA change were not retained in subsequent models testing moderation.

**Table 5:** Model variables and original AICs.

	Int	Cond	ΔNA	ΔPA	SA	SA x ΔNA	Cope	Cope x ΔNA	Neuro	Neuro x ΔNA	Drink Import	Context Import	Context x ΔNA	Drink x ΔNA	AIC
Null	X														279.75
Model 1	X	X									X	X			268.22
Model 2	X	X	X	X							X	X			271.21
Model 3	X		X	X							X	X			263.36
Model 4	X		X								X	X			260.84
Model 5	X		X		X	X					X	X			264.62
Model 6	X		X				X	X			X	X			265.41
Model 7	X		X						X	X	X	X			260.00
Model 8	X		X						X	X	X	X	X		263.70
Model 9	X		X						X	X	X	X		X	262.47

Note. Models above the thin line were included in phase 1 of model selection (models 1-4) and models above the bold line and below the thin line (models 5-7) included moderation variables in phase 2 to find a final best fitting model. Models below the bold line (models 8 and 9) were follow-up models. Int=intercept; Cond=condition; SA=social anxiety; Cope=drinking to cope motives; Neuro=neuroticism; Drink Import=importance participants placed on receiving their drink choice; Context Import=importance participants placed on receiving their context choice.

**Table 6:** 95%CIs of bootstrapped AIC differences between models.

	Null	Model 1	Model 2	Model 3	Model 4	Model 5	Model 6	Model 7	Model 8	Model 9
Null	-									
Model 1	[25.29,26.56]	-								
Model 2	[29.30,30.62]	[3.31,4.76]	-							
Model 3	[29.53,30.82]	[3.54,4.96]	[-0.52, 0.95]	-						
Model 4	[29.16,30.43]	[3.17,4.57]	[-0.89, 0.56]	[-1.09,0.33]	-					
Model 5	[30.56,31.80]	[4.57,5.94]	[0.50,1.93]	[0.31,1.70]	[0.69,2.08]	-				
Model 6	[30.77,32.07]	[4.77,6.22]	[0.71,2.20]	[0.51,1.97]	[0.90,2.35]	[-0.47, 0.95]	-			
Model 7	[37.01,38.27]	[11.02,12.42]	[6.96,8.41]	[6.76,8.17]	[7.15,8.55]	[5.77,7.15]	[5.50,6.94]	-		
Model 8	[37.28,38.58]	[11.28,12.72]	[7.23,8.71]	[7.03,8.48]	[7.41,8.85]	[6.04,7.45]	[5.77,7.25]	[-0.43, 1.00]	-	
Model 9	[37.69,38.96]	[11.69,13.10]	[7.63,9.09]	[7.43,8.86]	[7.82,9.24]	[6.45,7.84]	[6.18,7.63]	[-0.02, 1.37]	[-0.33,1.12]	-

Note. Like in Table 5, lines denote phases of model selection accordingly. All model differences are such that the row model is subtracted from the column model (e.g., null model AICs – model 1 AICs, null model AICs – model 2 AICs going down the null model column) such that negative CIs denote that the column model is better (produces smaller AICs) than the row model while positive CIs denote that the column model is not better (produces larger AICs) than the row model.

#### Step 2: Moderation models – second hypothesis

To test for moderation, variables (i.e., neuroticism, drinking to cope, and social anxiety) and interaction terms (e.g., NA change score x neuroticism) were added to model 4 (the new base model) for further comparison (see Table 5). Results from the 95% CIs showed that all three moderation models produced smaller AICs and thus, a better fit to the data, than the base model that included NA change. Additionally, model 7 produced smaller AICs than both models 5 and 6. As such, model 7 (which contained neuroticism and a neuroticism x NA change interaction term and is hereafter referred to as the neuroticism model) was retained as the best fitting model. This model was then compared to one that also included a context importance by NA change interaction term, as well as a model that included a drink importance by NA change interaction (models 8 and 9). The 95% CIs of AIC differences showed that models 8 and 9 were not consistently better fitting than the neuroticism model (see Table 6). Given these inconsistencies and because the neuroticism model is a simpler model, it was retained as the final model and investigated further.

#### Model exploration – neuroticism model results

The variables in the neuroticism model (i.e., NA change, neuroticism, neuroticism by NA change, drink importance, and context importance) were submitted to 5000 bootstraps with resampling to obtain subsequent coefficients comparing the log odds of drinking alcohol socially to the reference outcome of drinking alcohol alone. Coefficients comparing drinking alcohol alone to non-alcohol contexts were not examined given that so few participants chose non-alcoholic beverages. The 95% CIs of these bootstrapped coefficients were also obtained (see Table 7).

**Table 7:** Coefficients based on the original data and bootstrapped 95%CIs.

	Model 7	
	Original B (SE)	Bootstrapped 95% CI
Intercept	1.38 (1.80)	-
NA Change	-0.06 (0.61)	[-1.50,1.55]
Neuroticism	0.01 (0.03)	[-0.05,0.07]
Neuroticism x NA Change	0.00 (0.01)	[-0.03,0.04]
Drink importance	0.16 (0.19)	[-0.27,0.54]
Context importance	-0.87 (0.20)	[-1.24,-0.35]

The stepwise feature selection procedure produces a final model with good fit to the data and thus all variables included contribute to the variance in the outcome and are important (e.g., [45]). However, individually, these variables may be weaker or stronger in their contribution to explaining the outcome variance. Results from both the original data and bootstrapping indicated that most variables, with the exception of context importance, were weakly associated with outcome choice individually, such that the coefficients were generally very small, and the 95% CIs centered around zero (see Table 7).

**Fig 2:**
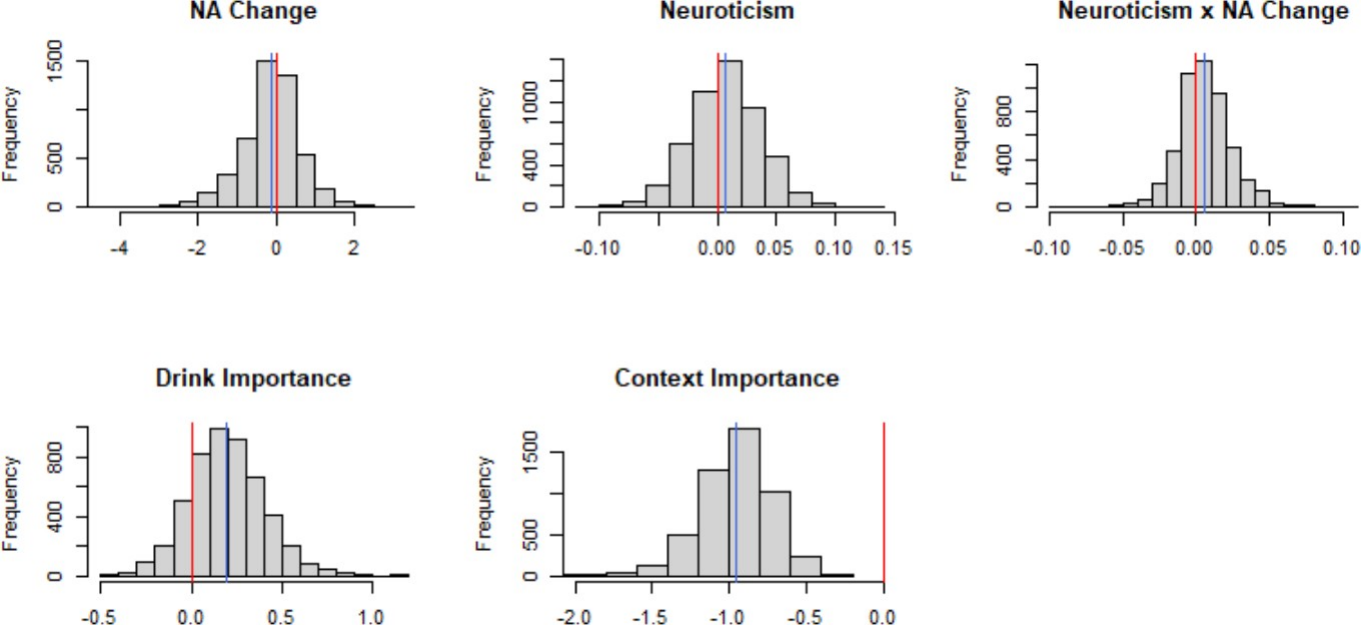
Histograms of the distribution of the bootstrapped coefficients. Blue line=mean of bootstrapped coefficients; red line=zero.

Further investigations into the associations between these variables and outcome choice were done through the generation of histograms of the bootstrapped coefficients (see Fig 2) as well as calculating z-scores for the distance between the mean of the bootstrapped coefficients and zero, and percentages of the bootstrapped coefficients above or below zero (see Table 8). These investigations further supported findings of a weak contribution of most of the variables in explaining outcome choice, but the directions of the effects were generally in the anticipated directions. Specifically, more than half of the bootstrapped coefficients of the NA change variable were negative, and zero was 0.18 standard deviations (SDs) to the right of the mean of the bootstrapped coefficient (see Table 8). These results suggest that the coefficient of NA change was negative, such that with increases in NA change, there was a decreased log odd of choosing to drink alcohol socially compared to alcohol alone.

**Table 8:** Bootstrapped means of coefficients, z-scores of 0 from the means and the percentage of bootstrapped coefficients out the 5000 coefficients that are above or below zero.

	Mean of coefficients	SDs of 0 from mean	Coeffs above/below 0
NA Change	-0.14	0.18	56% below 0
Neuroticism	0.01	-0.24	60% above 0
Neuroticism x NA Change	0.01	-0.32	62% above 0
Drink Importance	0.19	-0.94	83% above 0
Context Importance	-0.95	4.20	100% below 0

Evidence from bootstrapping also suggested a positive coefficient for both neuroticism and neuroticism by NA change. The calculated z-scores indicated that zero was 0.24 SDs and 0.32 SDs to the left of the mean bootstrapped coefficient of neuroticism and neuroticism by NA change, respectively, and the majority of the bootstrapped coefficients of both were above zero (see Table 8). In the case of neuroticism, this indicates that increases in neuroticism increased the log odds of choosing to drink alcohol socially versus alone, though these results should be considered in the context of the interaction between neuroticism and NA change (described below).

**Fig 3:**
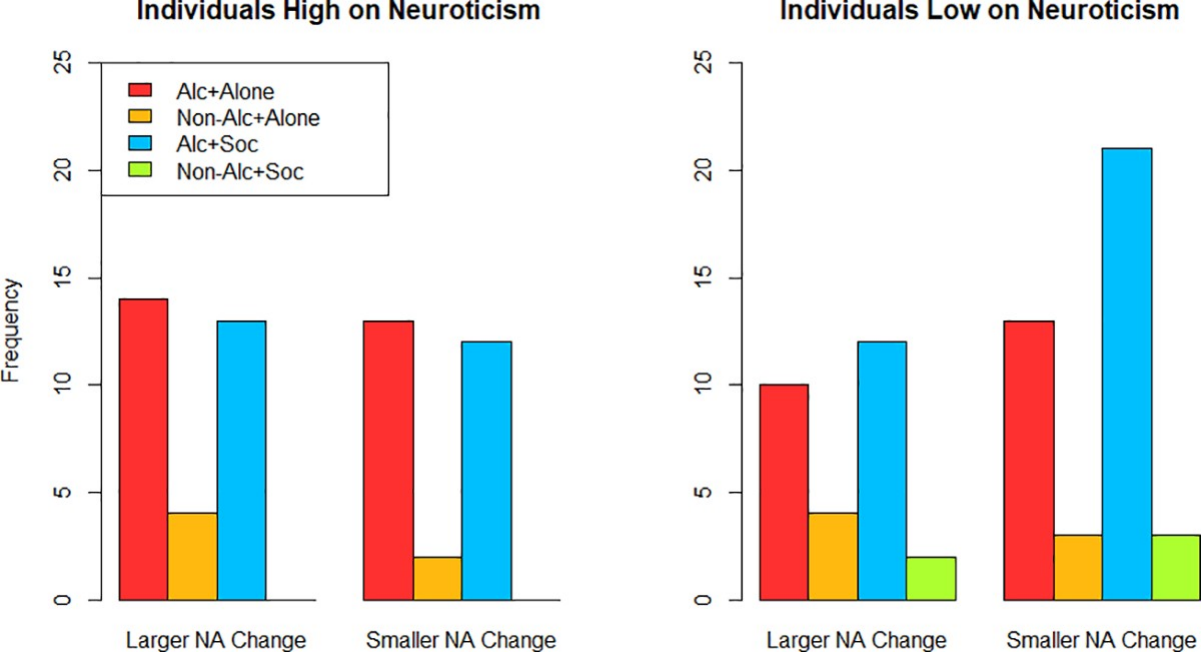
Bar plots investigating interaction between NA change and neuroticism.

Post hoc analyses were conducted to understand the interaction between neuroticism and NA change on outcome choice. Median splits of these variables were generated based on the original data, and frequencies of the outcome choices for individuals experiencing smaller and larger changes in NA across low and high neuroticism scores were examined with bar plots (see Fig 3). The bar plots suggested that outcome choice was not appreciably different for individuals high in neuroticism across smaller and larger NA change scores; in both cases there were slightly more individuals who chose to drink alcohol alone than socially. For individuals low in neuroticism, there were more individuals who chose to drink alcohol socially than alcohol alone, but those who experienced relatively smaller increases in NA were more likely to choose to drink alcohol socially than individuals who experienced relatively larger increases in NA.

Bootstrapping of the importance variables suggested a weak association between drink importance on outcome choice with bootstrapped coefficients suggesting a positive coefficient. That is, the bootstrapped coefficients for drink importance were mostly positive and zero was almost a full SD to the left of the mean (see Table 8). This suggests that increases in placing importance on receiving one’s drink choice were associated with increased log odds of choosing to drink alcohol socially relative to drinking alone, but the association was weak.

The association between context importance and outcome choice looked largely negative and unlike the other variables, the 95% CI did not contain zero. Additionally, the histogram, z-score of 0 from the mean, and percentage of coefficients below zero further showed this variable strongly contributed to explaining the variance of outcome choice, such that increases in context importance were associated with a decreased log odds of choosing alcohol socially over alcohol alone (see Table 8). This was not qualified by an interaction with NA change given the model selection did not retain a model with this term.

To be thorough, we also ran several alternative models to determine whether our final best fitting model was not retained solely because of the influence of the importance variables which were included from the outset as control variables (i.e., we aimed to determine whether the reason for improved model fit across successive steps of model selection was not solely due to the inclusion of the importance variables). To this end, we ran a model which only contained the two importance variables, and we re-ran all of the aforementioned analyses without the importance variables included as controls, but rather including them only as moderator variables. None of these models were a better fit to the data than the final best fitting model.

## Discussion

Solitary drinking is a risky drinking pattern associated with increased alcohol consumption and varied psychosocial problems (e.g., [6,7,12]). It also prospectively predicts later alcohol use and consequences even after accounting for earlier risk factors (e.g., [6,12]), suggesting that it may be an early warning sign for the development of AUD. Because of this, there is interest in why individuals engage in this behavior, with particular focus on a negative reinforcement mechanism whereby individuals drink alone to alleviate NA (e.g., [5,15,17]). However, studies testing this hypothesis have been correlational in nature. In an endeavor to fill this gap, the present study experimentally tested whether NA (versus positive or no affect change) would increase the preference to drink alcoholic beverages (versus nonalcoholic beverages) alone (versus with an ostensible other participant) in a sample of young adults with a history of drinking alone, and whether this association would be stronger for individuals higher in theoretically-relevant factors (i.e., neuroticism, drinking to cope motives, and social anxiety).

The first hypothesis was not supported, as condition assignment did not influence outcome choice, based on AIC comparisons of models that did and did not include the condition variables. There was mixed evidence, however, for the success of the mood manipulation. As expected, participants in the NA condition experienced a significant increase in NA, even after controlling for baseline NA scores. However, participants in the control condition also experienced a significant increase in NA, and participants in the PA condition did not experience a significant increase in PA. Had there been a clean distinction between the conditions on affect in the ways intended, this might have parsed out more variance in explaining outcome choice.

Given the mixed success of the mood manipulation, an NA change variable was created and used in analyses. This precludes causal arguments for a negative reinforcement mechanism behind solitary drinking, but still informs our understanding of the relationship between NA and drinking alone. Unlike condition, NA change was included in the final best-fitting model. Though there was only a weak association between this variable and outcome choice, it supported prior research. That is, based on the distribution of the bootstrapped histograms, the coefficient was negative and indicated that increases in NA were associated with solitary alcohol consumption over social alcohol consumption. This supports prior research in which NA and related constructs (e.g., depression) have shown positive associations with solitary drinking (e.g., [7,11,12,46]). Similarly, PA change was not retained in a final model suggesting that it has less importance in influencing solitary drinking behavior. This is also backed by prior literature; for instance, solitary drinking is not associated with PA in adults [47], nor is it associated with drinking in the context of PA among adolescents [12].

While the association between NA change and increased likelihood of choosing to drink alcohol alone in the current study is consistent with prior research, the strength of this association was weak. Speculatively, it may be that despite statistically significant increases in NA, these changes were not strong enough to impact outcome choices. It is possible that the laboratory task used here did not heighten NA the way that real world experiences do, which produces stronger associations between NA and solitary drinking (e.g., [12,14,22]). That is, negative life events and situations in natural settings may generate more immediate and intense reactions and have greater import on behavior than tasks in artificial laboratory contexts, and thus, the effect of NA on outcome choice in the current study was not as strong as would be expected. More research is indicated, particularly experimental studies with more robust mood manipulations capable of inducing stronger NA (e.g., social rejection paradigms; [48], although this paradigm poses its own challenges because participants would likely choose to drink alone as a way to avoid drinking with those who socially rejected them). Future studies that focus on individuals high on trait neuroticism might also be beneficial, as these individuals would likely experience larger increases in negative mood in response to laboratory mood manipulations and perhaps would be more inclined to choose to drink alone.

A second aim of the study was to examine whether individual differences in neuroticism, drinking to cope motives, and social anxiety would magnify the relationship between NA and choosing to drink alcohol alone. Findings indicated that only neuroticism and its interaction with NA change influenced preference to drink alcohol alone, albeit weakly. Specifically, post hoc analyses showed that those higher in neuroticism preferred to drink alcohol alone more than alcohol socially, while those lower in neuroticism preferred to drink alcohol socially more than alone, and among these latter individuals, this difference was even more pronounced for participants who experienced relatively smaller changes in NA. These findings support the current literature on solitary drinking to a degree. The increased preference of individuals high in neuroticism to choose to drink alcohol alone aligns with prior research on related constructs and their associations with solitary drinking (e.g., positive associations between NA experiences/traits and solitary drinking; e.g., [46]). However, the post hoc findings also showed that increases in NA did not lead to increased choices of solitary alcohol consumption over social alcohol consumption for those high in neuroticism—in fact, there was not a discernable difference in choices across smaller and larger changes in NA in these individuals. This is counter to prior research by Mohr and colleagues (2001) who found that the association between experiencing NA and solitary drinking is stronger for individuals high in neuroticism [26]. For those who were low in neuroticism, results showed that experiencing smaller changes in NA led to an increase in the likelihood to choose to drink alcohol socially. These findings may suggest that those lower in neuroticism are generally unlikely to drink alcohol alone, though they become more likely to choose solitary drinking contexts when experiencing acute increases in NA. Again, though, the size of the effect was quite small and these results should be considered preliminary at best.

Surprisingly, the importance participants placed on getting their drink and context choices was associated with their preferences for drinking alcohol alone versus socially. Increases in the importance placed on receiving one’s drink choice were associated with increased log odds of choosing social alcohol consumption over solitary alcohol consumption, while increases in importance placed on receiving one’s context choice was associated with decreased log odds of this, and this latter variable was strongly associated with outcome choice. It is unclear why drink importance would be associated with choosing to drink alcohol socially over alone, but one potential reason may be the normative nature of social drinking among this demographic. While this study included individuals with a history of drinking alone, these individuals still drank a majority of the time with other individuals. On average, 44% of their drinking episodes were in solitary settings, which means that more than half of their drinking episodes occurred in social settings. Thus, when controlling for NA change, social drinking may be the preferred method of drinking for these individuals.

The strong association between the importance placed on receiving one’s context choice and choosing to drink alone is also puzzling. This association was not qualified by an interaction with NA change, given that a model that included this interaction term was not a better fitting model than one without, which indicates that this association was not stronger for those who experienced increased NA after the mood manipulation. Bivariate correlations showed that context importance was positively correlated with social anxiety, neuroticism, and drinking to cope motives, though. While speculative, it may be that the importance placed on getting one’s context choice is indicative of an underlying tendency to experience negative affectivity and social discomfort, and individuals with this tendency are more inclined to drink alcohol solitarily. However, the context importance variable was not related to the amount of time spent drinking in solitary contexts in the past year. More research is needed to explore this surprising finding.

This study has limitations. The most important limitation was the mixed results of the mood manipulation in eliciting the appropriate change in affect (or lack of change in affect) in the designated conditions. There was an increase of NA in the control condition, and no significant increase in PA in the PA condition. This was surprising, as the manipulation was successful in pilot testing. Regardless, the manipulation was not wholly successful in the present study. Control condition participants may have experienced NA because receiving no feedback left them uncertain of their performance, which may have been a source of NA itself. Indeed, some researchers propose that uncertainty is a subjective experience of anxiety [49]. In the case of the PA condition, it might have been that the manipulation was not a powerful enough induction or that participants did not believe the positive feedback they received. We did not assess for these variables, however, so these explanations are speculative.

Another limitation is that the study is hindered by the artificial context of the experimental setting. Drinking alcohol in a laboratory environment is different from participants’ typical drinking experiences, and their choices may have been affected by this setting. Similarly, asking individuals about their drink and room choices outright may also have affected their decisions given that these direct questions are not how individuals would normally make their drinking decisions. Future studies may want to use more covert ways of having individuals choose their beverage and drinking context, as well as potentially allow for consumption in rooms with a more “natural” ambiance for drinking. Further, we recruited individuals who reported solitary drinking, but future studies might consider additional eligibility criteria including heavy drinking status and scoring highly on trait neuroticism, both of which might increase the likelihood of choosing to drink alone in the context of a negative mood induction. This study focused on young adults, and future studies are indicated to explore mechanisms for solitary drinking among older individuals (see [50]). Finally, it is important to note that individuals who volunteer to participate in laboratory alcohol studies may not be representative of all drinking individuals.

Despite these limitations, the study design was methodologically strong. The use of a familiarity task helped to circumvent potential confounding effects of participants’ choosing solitary drinking just to avoid being with a stranger, and strengthens the claim that individuals honestly preferred to drink alone when they chose this option. Related, while individuals predominantly picked alcoholic over non-alcoholic beverages, the possibility of having either option was useful, as it allowed for claims that individuals preferred alcohol over non-alcoholic beverages. Further, rather than hypothetical choices, participants believed that their choices had real consequences, strengthening the confidence we had in measuring their true preferences. The use of a more nuanced statistical method in lieu of traditional frequentist techniques was also a strength. Given the binary distinction of p-values and significance testing, employment of this method would have masked the contribution of variables and rendered the majority of them “null”. Thus, erroneous claims that NA did not influence actual drinking context preference would be made. Finally, the study design, hypotheses, and analysis plan were preregistered before the start of data collection.

In sum, the present study did not provide evidence for a causal role of NA in driving solitary drinking among young adults. However, this experimental lab study did provide further evidence that NA plays a role in this risky form of drinking behavior. Continued experimental research, which tests specific causal mechanisms underlying solitary drinking is needed. Understanding why certain individuals drink alone, and who is most at risk for solitary drinking behavior, will help to develop effective treatment options for specific subsets of individuals who are at-risk for developing drinking problems.

## Supporting information

S1 File Raw dataset used in analyses. (SAV)

S2 File Bootstrapped AIC values per model. (CSV)

S3 File Bootstrapped coefficients from the neuroticism model. (CSV)
